# Junction formation and current transport mechanisms in hybrid n-Si/PEDOT:PSS solar cells

**DOI:** 10.1038/srep13008

**Published:** 2015-08-17

**Authors:** Sara Jäckle, Matthias Mattiza, Martin Liebhaber, Gerald Brönstrup, Mathias Rommel, Klaus Lips, Silke Christiansen

**Affiliations:** 1 Institut Nanoarchitekturen für die Energieumwandlung, Helmholtz-Zentrum Berlin für Materialien und Energie GmbH, Hahn-Meitner-Platz 1, 14109 Berlin, Germany; 2 Christiansen Research Group, Max-Planck-Institute for the Science of Light, Günther-Scharowsky-Str. 1, 91058 Erlangen, Germany; 3 Energy Materials In-Situ Laboratory Berlin (EMIL), Institut für Silizium-Photovoltaik, Helmholtz-Zentrum Berlin für Materialien und Energie GmbH, Kekuléstrasse 5, 12489 Berlin, Germany; 4 Fraunhofer Institut für Integrierte Systeme und Bauelementetechnologie IISB, Schottkystrasse 10, 91058 Erlangen, Germany

## Abstract

We investigated hybrid inorganic-organic solar cells combining monocrystalline n-type silicon (n-Si) and a highly conductive polymer poly(3,4-ethylenedioxythiophene)-poly(styrene sulfonate) (PEDOT:PSS). The build-in potential, photo- and dark saturation current at this hybrid interface are monitored for varying n-Si doping concentrations. We corroborate that a high build-in potential forms at the hybrid junction leading to strong inversion of the n-Si surface. By extracting work function and valence band edge of the polymer from ultraviolet photoelectron spectroscopy, a band diagram of the hybrid n-Si/PEDOT:PSS heterojunction is presented. The current-voltage characteristics were analyzed using Schottky and abrupt pn-junction models. The magnitude as well as the dependence of dark saturation current on n-Si doping concentration proves that the transport is governed by diffusion of minority charge carriers in the n-Si and not by thermionic emission of majorities over a Schottky barrier. This leads to a comprehensive explanation of the high observed open-circuit voltages of up to 634 mV connected to high conversion efficiency of almost 14%, even for simple planar device structures without antireflection coating or optimized contacts. The presented work clearly shows that PEDOT:PSS forms a hybrid heterojunction with n-Si behaving similar to a conventional pn-junction and not, like commonly assumed, a Schottky junction.

In recent years the promising combination of organic and inorganic materials, often termed hybrids, has led to the emerging research field of hybrid optoelectronic devices. Especially hybrid solar cells combining the now commercially available highly conductive polymers with established but also emerging inorganic semiconductor materials have triggered intensive research. Starting from different simple conjugated polymers like derivatives of polyaniline or polyacetylene by far the highest conductivity has been reached by polytiophenes, in particular poly(3,4-ethylenedioxythiophene) (PEDOT)^1^,^2^. Especially in a complex with poly(styrene sulfonate) (PSS) which acts as a charge counter balance to the oxidized PEDOT backbone during polymerization, highly doped states can be achieved^3^. This makes the ‘metal-like’ polymer PEDOT a very efficient hole transporter with a transmission window in the visible spectral range^4^. Moreover, by the addition of PSS a stable micro dispersion can be realized in water, making it an easy-to-process solution^3^. In a previous study we were able to show that optimizing the transport properties in PEDOT:PSS films, by adding the organic solvent dimethyl sulfoxide (DMSO), known in the literature as ‘secondary doping’^5^,^6^, is essential for achieving highly efficient hybrid solar cells^4^.

For various hybrid photovoltaic device concepts PEDOT:PSS has been combined with common inorganic semiconductors such as silicon or gallium arsenide but is also used as a hole extraction layer for the comparably new perovskite solar cells^4^,^7^,^8^,^9^. One promising approach are cells with a type (iii) hybrid interface^7^ consisting of a transparent highly conductive polymer and an absorbing inorganic semiconductor, thereby permitting an efficient charge separation and charge transport. Photovoltaic devices combining PEDOT:PSS and crystalline silicon have first been proposed in 2010^7^. In past years planar hybrid n-Si/PEDOT:PSS junctions have reached efficiencies beyond 12%^10^,^11^. By improving passivation, contacting, and back junction formation as well as by including a thin tunneling oxide layer in the hybrid interface, Zielke *et al.* were even able to realize efficiencies around 17%, with the potential to reach 22%^12^. This device concept has also been implemented on nanostructured silicon substrates, however so far with lower efficiencies^13^.

Despite the enormous success of this device concept, the working principal has so far not been completely resolved. For instance, the interface between the highly doped PEDOT:PSS and silicon, which is responsible for charge separation, was mostly treated as a Schottky junction^7^,^13^,^14^,^15^,^16^, assuming that the polymer has ‘quasi-metallic’ behavior. Erikson and co-workers were able to show for hybrid n-Si/PEDOT:PSS field effect transistors with differently doped silicon substrates, that the created built-in potential is so high, that an inversion layer forms at the surface of the silicon substrate^17^. In a recent study^10^ we have pointed out that the strong dependence of the open circuit voltages (*V*_*oc*_) on the doping concentration of the silicon substrate observed for hybrid n-Si/PEDOT:PSS solar cells cannot be explained by assuming a Schottky junction. Already in the 1990s Sailor *et al.* suggested for a similar hybrid system, highly doped poly-(CH_3_)_3_Si-COT/n-Si, a bulk-diffusion limited *V*_*oc*_ but still assuming a Schottky junction^15^. Price *et al.* showed the advantage of a silicon junction with PEDOT:PSS for solar cells compared to a junction with gold. They assumed a Schottky junction but concluded that a very low thermionic recombination velocity of the electrons from the silicon into PEDOT:PSS is responsible for the high *V*_*oc*_ values^7^. Furthermore, results from different approaches using conducting polymers as interlayers between carbon nanotubes or other polymer/Si material systems state a similar enhancement of *V*_*oc*_ and power conversion efficiency (PCE)^18^,^19^,^20^,^21^. Recently published studies show highly efficient n-Si/PEDOT:PSS solar cells, with dark saturation current densities being magnitudes smaller than expected for a Schottky junction^11^,^22^.

In the present study the junction and the device performance of hybrid planar n-Si/PEDOT:PSS photovoltaic cells are investigated in great detail. Following our previous work, where we have pointed out that a solar cell based on the spin coated highly conductive polymer PEDOT:PSS and n-type silicon does not show the characteristics of a majority carrier driven Schottky junction^10^, the scope of our present work is to prove that this hybrid interface can be described by a minority carrier driven conventional pn-heterojunction instead. For this, we extract junction parameters and solar cell characteristics of hybrid devices based on silicon substrates with different doping concentrations. We use current density-voltage (dark J-V) and small signal capacitance-voltage (C-V) measurements as well as the photovoltaic response (illuminated J-V) to extract the solar cell parameters. We compare our experimental findings with predictions of minority carrier drift-diffusion and Schottky junction models. In addition, ultraviolet photoelectron spectroscopy (UPS) is used to complete our findings and establish a band diagram for the hybrid n-Si/PEDOT:PSS junction.

## Junction models

There are two approaches to describe the junction between a moderately doped n-type semiconductor and a highly doped p-conducting ‘metal-like’ organic layer. One is based on the Schottky junction theory that explains the interface of a semiconductor to a metal. The other is the description of a one-sided abrupt junction between a moderately doped n-type semiconductor region and a highly doped p-type semiconducting region. For both cases the current density-voltage characteristic of such a photovoltaic junction is in the simplest form described by the ideal diode equation under illumination (Equation 1)^23^. Rewriting Equation 1 at open circuit conditions (*J* = 0) shows that the open circuit voltage *V*_*oc*_ of a solar cell mainly depends on the dark saturation current density *J*_0_ and the short circuit current density *J*_*sc*_, see Equation 2.


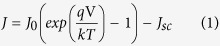



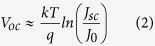



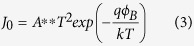



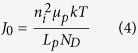


At a Schottky junction between a metal and a high-mobility semiconductor like silicon, the dominating transport mechanism is the thermionic emission of majority carriers over the potential barrier *ϕ*_*B*_ that forms at the interface^24^. Equation 3 describes *J*_0_ for a Schottky junction. A** denotes the reduced effective Richardson constant including effects of tunneling and scattering of majority carriers at phonons as well as a correction factor for a small contribution of majority carrier diffusion for moderately doped silicon. At room temperature, in a reasonably small applied field, A^**^ is about 110 A/(cm∙K)^2^ 25. Equation 3 shows that for a Schottky junction *J*_0_ mainly depends on the Schottky barrier height *ϕ*_*B*_. Ideally at a junction of a metal with an n-type semiconductor *ϕ*_*Bn*_ is given by the difference between the metal work function *qϕ*_*M*_ and the electron affinity of the semiconductor *qχ*_*S*_, reduced by the attractive force between electrons in the semiconductor and induced positive image charges in the metal, described by the so-called Schottky-barrier lowering Δ*ϕ*^23^.


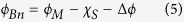


Therefore *ϕ*_*Bn*_ only very slightly decreases with higher doping concentration *N*_*D*_ of the inorganic semiconductor because 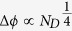
23. Following Equation 3, *J*_0_ should then slightly increase with higher *N*_*D*_, leading to a weakly decreased *V*_*oc*_ if *J*_*sc*_ stays constant (cf. Equation 2). At most common n-Si/metal junctions, *ϕ*_*Bn*_ is lower than expected from Equation 5 and becomes independent of the metal work function. This is due to Fermi level pinning at a high density of surface states located in the semiconductor band gap that are induced by the tunneling and decay of the metal electron wave functions into the semiconductor^26^. In this case *ϕ*_*Bn*_ is still independent of the doping concentration of the semiconductor. The Fermi level can also be pinned at a non-passivated free standing silicon surface^27^ and correspondingly in semiconductor heterojunctions.

In contrast to a Schottky diode, in a junction between a p-type and a n-type semiconductor the transport processes are dominated by the diffusion of minority carriers. Assuming that the doping profile between the two semiconductors changes abruptly at the junction the diode equation, Equation 1, can be given following Shockley^28^. If the doping of the p-type semiconductor is substantially larger than that of the n-type semiconductor, the dark saturation current density *J*_0_ of the so called one-sided abrupt p^+^n-junction is defined by Equation 4. In this *μ*_*p*_ denotes the mobility, *L*_*p*_ the diffusion length of the minority carriers (holes for n-Si) and *n*_*i*_ the intrinsic carrier concentration. *J*_0_ solely depends on properties of the moderately doped n-type semiconductor and is inversely proportional to its doping concentration *N*_*D*_. Therefore, following Equation 2, the *V*_*oc*_ in a p^+^n-junction should increase with larger *N*_*D*_. In the Shockley equation only diffusion, which is limited by bulk recombination mechanisms outside of the space-charge region, is considered. This is adequate only for semiconductors with large intrinsic carrier densities^23^. For silicon, with its low intrinsic charge carrier concentration, it is more suitable to additionally consider recombination and generation at traps inside the space-charge region^29^. By also including the area specific parallel *R*_*p*_ and serial resistance *R*_*s*_ of the device, the current density-voltage characteristics of an abrupt p^+^n-junction solar cell is then described by the two-diode model (Equation 6)^23^. In this implicit equation *J*_01_ corresponds to the dark saturation current density from bulk diffusion (cf. Equation 4) while *J*_02_ represents trap-assisted generation and recombination processes in the space-charge region.


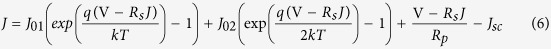


## Results and Discussion

**Figure 1:**
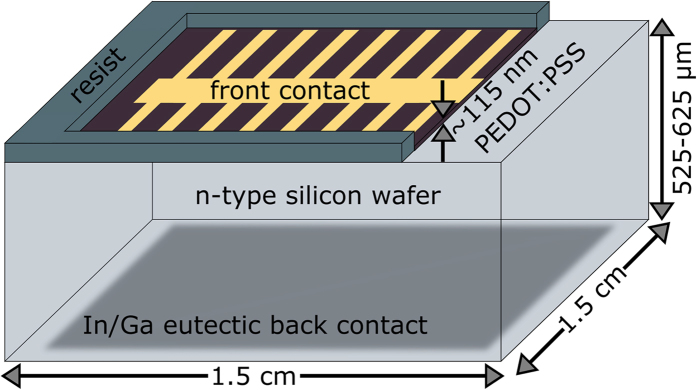
Schematic of the device structure of a fabricated n-Si/PEDOT:PSS solar cell. The surface of the monocrystalline n-type silicon wafer is structured by resist to define an active area for the spin coated polymer. The device is contacted by a top evaporated Au grid and a back side scratched In/Ga eutectic.

**Figure 2:**
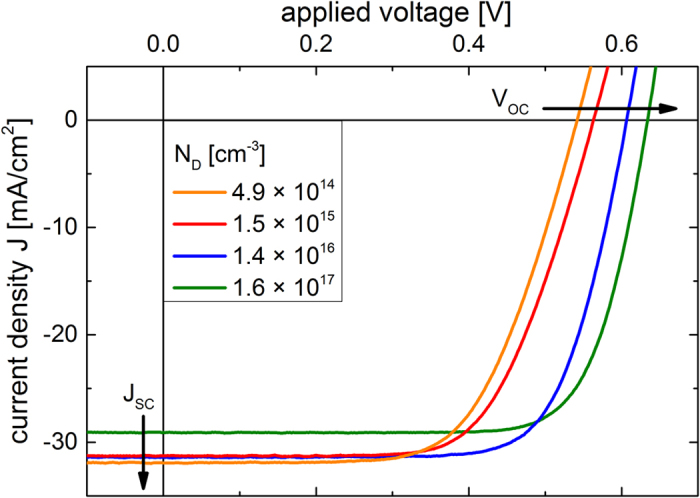
Photovoltaic properties of n-Si/PEDOT:PSS solar cells. J-V-characteristics under AM1.5 spectrum irradiation of the hybrid PV-devices with differently doped (*N*_*D*_) silicon substrates.

Hybrid n-Si/PEDOT:PSS solar cells, displayed in Fig. 1, based on four differently doped silicon substrates with *N*_*D*_ ranging between 10^14^ cm^−3^ and 10^17^ cm^−3^ have been fabricated. Fig. 2 illustrates the photovoltaic response of the four different solar cells illuminated through the polymer contact by an AM1.5 spectrum. All relevant solar cell parameters extracted from the illuminated J-V-curves are collected in Table 1. The hybrid solar cells presented show high efficiencies above 13%, as was previously achieved by other groups^12^,^30^. The short circuit current density *J*_*sc*_ is essentially constant at around ~31 mA/cm^2^ for moderate doping concentrations and only slightly decreases for the highest doped silicon substrate to 29.1 mA/cm^2^. The fill factor (FF) slowly increases with higher *N*_*D*_ from 64% to 75%. A reason for the high *J*_*sc*_ and FF of hybrid n-Si/PEDOT:PSS solar cells is the metallic character of PEDOT:PSS, as reported before^4^. In contrast to chromophores like P3HT, which have good absorption but modest conducting properties^31^,^32^, PEDOT:PSS is a highly conductive transparent polymer. Because of the high transparency, most of the light is absorbed in the silicon wafer, reflected in the high *J*_*sc*_ of the hybrid solar cells. Due to its high conductivity the polymer is able to transport holes very efficient, leading to a high FF. The outstanding performance of n-Si/PEDOT:PSS solar cells results in particular from the high *V*_*oc*_ values of up to 630 mV, that are comparable with conventional high-temperature emitter diffused solar cells^33^. As indicated by the arrow in Fig. 2, *V*_*oc*_ strongly increases with increasing doping concentration *N*_*D*_ of the silicon substrates. Following the conclusions in our recent publication^10^, the increase of *V*_*oc*_, while *J*_*sc*_ is almost constant or even decreasing, has to have its origin in a decreasing *J*_0_ with increasing *N*_*D*_ (cf. Equation 2).

As addressed in the introduction the hybrid interface between the highly conductive ‘metal-like’ polymer PEDOT:PSS and silicon is commonly assumed to be a Schottky junction^7^,^13^,^16^. In this case *J*_0_ mainly depends on the barrier height *ϕ*_*Bn*_ (cf. Equation 3). *ϕ*_*Bn*_, as it is defined in Equation 5, can also be expressed in terms of the built-in potential ψ_bi_, describing the band bending due to Fermi level alignment at the interface^23^.


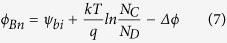


**Figure 3:**
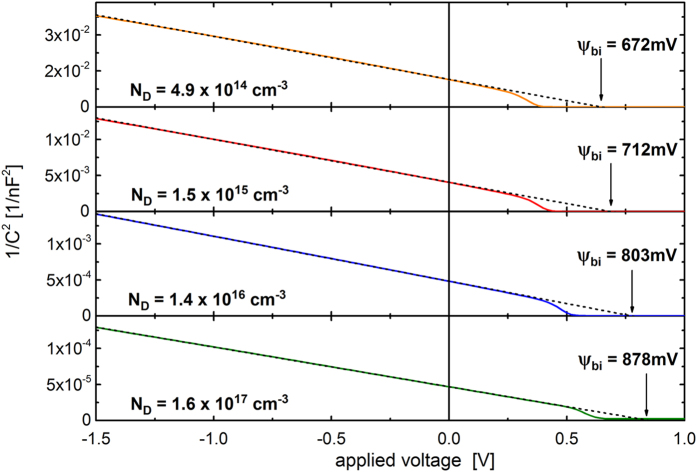
C-V characteristics of n-Si/PEDOT:PSS junctions. 1/C^2^-V plots for differently doped silicon substrates. The built-in voltage *ψ*_*bi*_ is extracted from the V-axis intercept of the extrapolation of the linear part of the data while the silicon substrate doping concentration *N*_*D*_ is given by the slope of the linear fit.

**Figure 4:**
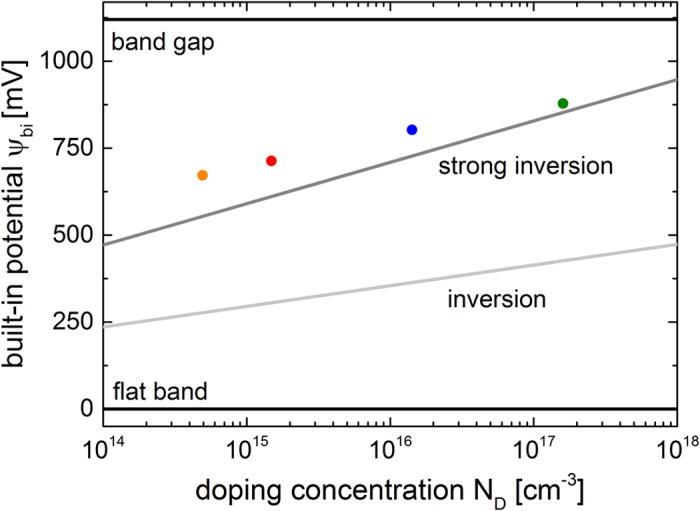
Inversion at the n-Si/PEDOT:PSS interface. Built-in potential *ψ*_*bi*_ (apapted from Fig. 3) for differently doped silicon substrates with the treshold values for inversion and strong inversion.

The second term in Equation 7 denotes the difference between the Fermi level and electron affinity of the silicon substrate where *N*_*C*_ is the effective density of states in the conduction band. To determine the barrier height at the n-Si/PEDOT:PSS junctions the capacitive response (C-V) of the solar cells was investigated. While with applying a forward voltage to a rectifying junction the diffusion capacitance from injected charges starts to be dominant, with increasing reverse voltage the decreasing capacitance of the depletion layer capacitance can be observed^23^. Plotting 1/C^2^-V allows the determination of *ψ*_*bi*_ at the junction and *N*_*D*_ of the silicon substrate from the progression of the depletion layer capacitance,


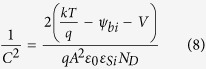


where A is the diode area and *ε*_0_*ε*_*Si*_ the permittivity of silicon. Fig. 3 depicts the characteristic 1/C^2^-V plots for differently doped n-Si/PEDOT:PSS devices. The linear parts are fitted and extrapolated to obtain *ψ*_*bi*_ from V-axis intercepts and *N*_*D*_ from the slopes, relating to Equation 8. The derived doping concentrations for all differently doped silicon are very well in the range of values given by the wafer manufactures. *ϕ*_Bn_ is calculated following Equation 7. All values extracted from the capacitance measurements are summarized in Table 1. As expected from Equation 5 and 7 *ψ*_*bi*_ increases with increasing *N*_*D*_ while *ϕ*_*Bn*_ stays constant. Following Equation 5 the work function of PEDOT:PSS (*qϕ*_*P*_ = *qϕ*_*M*_) can be calculated, with the electron affinity of silicon being *qχ*_*S*_ = *4.05* *eV*^23^. *qϕ*_*P*_ between 5.00 eV and 5.06 eV are extracted from the data, which is in good agreement with literature values^34^,^35^,^36^. This would suggest a nearly ideal Schottky barrier height formation. Fermi level pinning at the defects of the silicon surface, which is usually observed at n-Si/metal Schottky junctions and often at heterojunctions with silicon, does not occur for this hybrid junction. Plotting the dependence of the extracted *ψ*_*bi*_ on *N*_*D*_ in Fig. 4 shows that due to the band bending the intrinsic energy level of silicon is forced to cross the Fermi level at the surface to the polymer, referred to as inversion. PEDOT:PSS even induces a strong inversion, *qψ*_*bi*_ > *E*_*g*_ − 2(*E*_*C*_ − *E*_*F*_), at the silicon surface for all four hybrid solar cells based on the differently doped substrates. The n-type silicon is completely inverted to a p-type silicon at the interface to the polymer without any additional doping, as also recently observed for hybrid n-Si/PEDOT:PSS field effect transistors^17^. It has been demonstrated that PEDOT:PSS does provide a surprisingly effective surface passivation for silicon^12^, but the exact passivation mechanism is still unknown.

**Figure 5:**
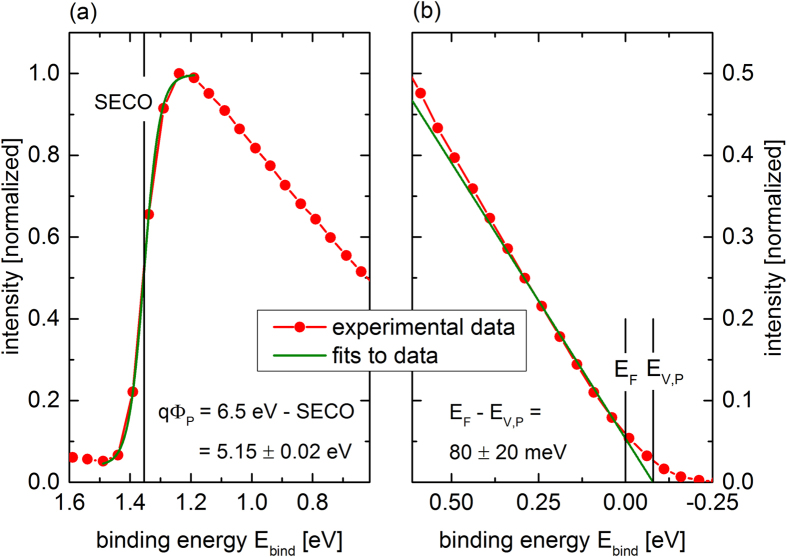
Ultraviolet photoelectron spectrum of a PEDOT:PSS film using 6.5 eV excitation energy. (**a**) Secondary electron cut-off (SECO) fittet by a Boltzmann sigmoid function for extraction of the work function *qϕ*_*P*_ and (**b**) valence band states near the Fermi level E_F_ with a linear extrapolation to the valence band edge E_V,P_.

The parameters extracted from the C-V measurements, the doping concentration *N*_*D*_ of the silicon substrate and the built-in potential *ψ*_*bi*_ of the hybrid junction in Fig. 3, can be used to construct a band diagram for the n-Si/PEDOT:PSS interface. While the material parameters for silicon are well known, the electron affinity being *qχ*_*S*_ = *4.05* *eV* and the band gap *E*_*g*,*Si*_(300 *K*) =1.12*eV*^23^, the energy levels of the polymer PEDOT:PSS depend on the particular used blend^37^, additives^35^ and post-treatment (heat, water absorption)^34^,^36^. For the PEDOT:PSS used in this study the work function *qϕ*_*P*_ as well as the position of the valence band edge *E*_*V*,*P*_ relative to the Fermi level *E*_*F*_ were determined by UPS measurements using an excitation energy of 6.5 eV. The UPS spectrum close to the secondary electron cut-off (SECO) and close to *E*_*F*_ is depicted in Fig. 5(a,b), respectively. The work function is calculated from the difference between the excitation energy 6.5 eV and the binding energy at the SECO. For this the drop of the photoemission is fitted by a standard Boltzmann sigmoidal fit where the center position is defined as the SECO, shown as a vertical black line in Fig. 5(a). The extracted work function of PEDOT:PSS is *qϕ*_*P*_ = 5.15 ± 0.02 *eV*. This is in good agreement with the values of 5.0 eV to 5.2 eV measured by other groups with UPS and Kelvin probe looking at similarly mixed and treated PEDOT:PSS^34^,^35^,^36^,^37^,^38^. *qϕ*_*P*_ extracted from UPS is slightly higher than the work function extracted from the C-V measurements (5.00–5.06 *eV*). While the capacitance-voltage measurements are carried out under ambient conditions after the complete device processing, the UPS is performed in vacuum on a separate, but identically in the ambient prepared, PEDOT:PSS layer on silicon. It has been shown before that residual or absorbed water from ambient air leads to a decreased work function compared to a vacuum dried polymer film^36^, possibly also explaining the slight difference observed here. The UPS spectra in Fig. 5(b) exhibits filled (valence) states up to the Fermi level, as expected for a highly p-doped polymer as PEDOT:PSS. Often the surface sensitive UPS spectra are dominated by the insulating PSS shell instead of by the conducting PEDOT:PSS grains^38^. In this study we used a low excitation energy (monochromized Xenon light at 6.5 eV). The inelastic mean free path of the generated photoelectrons is considerably larger than in the commonly used He-UPS^34^,^37^,^38^,^39^, as it is reflected in the universal curve^40^. This leads to a higher information depth of the UPS 6.5 eV spectra, so that a significant signal of PEDOT itself can be detected. Also in the binding energy range from 0 to 2 eV only PEDOT and not PSS should show a signal from filled valence states^39^. Therefore, the signal shown in Fig. 5 clearly corresponds to the density of valence band states (DOVS) of PEDOT itself. The position of the valence band edge *E*_*V*,*P*_ relative to the Fermi level *E*_*F*_ (*E*_*bind*_ = 0) was obtained by linear extrapolation of the DOVS leading edge to zero (cf. Fig. 5(b)). The determined valence band edge *E*_*V*,*P*_ lies 80 ± 20 *meV* above *E*_*F*_. Bubnova *et al.* recently showed a similar UPS spectrum for the highly conductive PEDOT:Tos also showing a large DOVS at *E*_*F*_^37^. As suggested before this could be traced back to the bipolaron band merging with the valence band at these high doping levels^41^. For p-doping the bipolaron bands are empty and this would lead to a Fermi level position below the valence band edge^42^.

**Figure 6:**
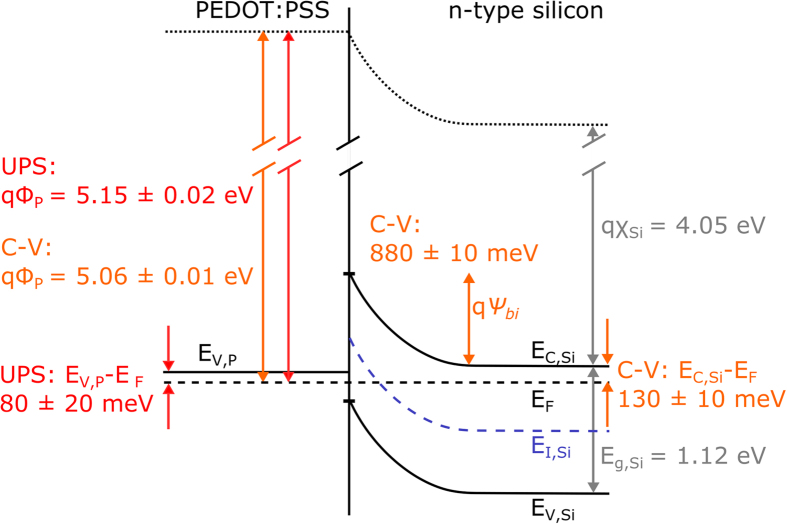
Junction formation at hybrid n-Si/PEDOT:PSS interfaces. Schematic of the band structure for a silicon bulk doping concentration of *N*_*D*_ = 1.6 × 10^17^ cm^−3^ using values extracted from capacitance (C-V), UV photoelectron spectroscopy (UPS) measurements and literature data^23^.

Combining the work function *qϕ*_*P*_ and the valence band edge position *E*_*V*,*P*_ (relative to *E*_*F*_) of PEDOT:PSS determined from UPS with the build-in voltage *ψ*_*bi*_ and the position of the Fermi level relative to the conduction band in silicon *E*_*C,Si*_ − *E*_*F*_ calculated by *N*_*D*_ (cf. second term in Equation 7) from the C-V measurements, a band diagram for the n-Si/PEDOT:PSS junction can be obtained. Fig. 6 shows the band diagram for the hybrid junction based on a silicon substrate doping of *N*_*D*_ = 1.6 × 10^17^ cm^−3^. The illustrated values show that *ψ*_*bi*_ forms ideally at the junction as the difference between the work function of PEDOT:PSS *qϕ*_*P*_ and silicon *qχ*_*S*_ + (*E*_*C,Si*_ − *E*_*F*_). This leads to an inversion of the silicon at the surface, embossed by a crossing of the Fermi level *E*_*F*_ by the intrinsic Fermi level *E*_*I,Si*_. As already illustrated in Fig. 4 the silicon is even strongly inverted by the polymer for all doping concentrations.

**Figure 7:**
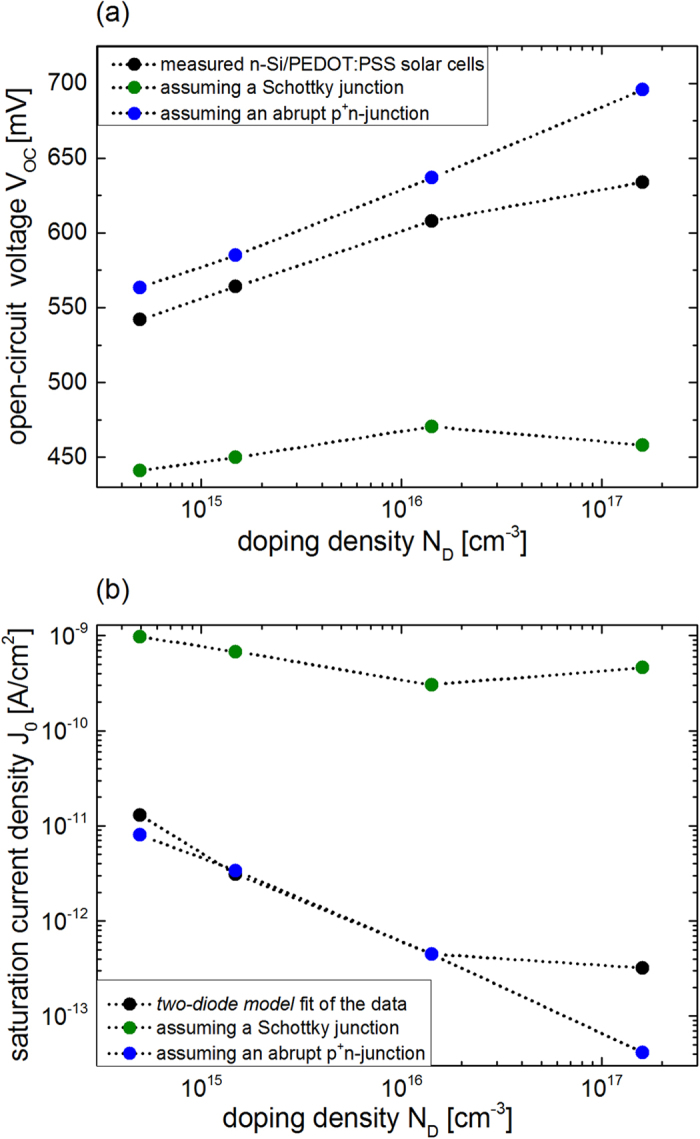
Dependence of (a) measured and calculated *V*_*oc*_ and (b) fitted and calculated *J*_0_ on the silicon substrate doping concentration *N*_*D*_ for n-Si/PEDOT:PSS solar cells.

With *ϕ*_*Bn*_ (Table 1) extracted from the capacitance-voltage measurements, the dark saturation current density *J*_0_ (Equation 3) and subsequently the open-circuit voltage *V*_*oc*_ (Equation 2) can be assessed assuming a Schottky junction. The calculated *J*_0_ and *V*_*oc*_ are collected in Table 2 and depicted in Fig. 7 (green dots). *J*_0_ is in the range of 1 nA/cm^2^ to 0.1 nA/cm^2^. As expected from the almost constant *ϕ*_*Bn*_ *J*_0_ shows only a slight variation with *N*_*D*_. With a constant *J*_*sc*_, this leads also to only slightly varying calculated *V*_*oc*_ between 441 mV and 470 mV (cf. Fig. 7(a)). Even though there is no Fermi level pinning at the silicon surface and the band bending leads to a strong inversion of the silicon, as discussed before, the calculated *V*_*oc*_ is still considerably smaller than the *V*_*oc*_ measured for the hybrid n-Si/PEDOT:PSS solar cells (cf. black dots in Fig. 7(a)). Also the distinct increase of the measured *V*_*oc*_ with increasing *N*_*D*_ is not reflected by the *V*_*oc*_ values calculated assuming a Schottky junction. This manifests the assumption that the thermionic emission of majority carriers cannot be the dominant transport mechanism in hybrid n-Si/PEDOT:PSS solar cells, as pointed out in our previous work^10^.

Therefore, the hybrid n-Si/PEDOT:PSS interface is instead assumed to be a pn-junction, where the dominant transport mechanism is the diffusion of minority carriers. Following the UPS measurements showing the high doping of the polymer in the previous section, the interface is described by a one-sided abrupt p^+^n-junction, where *J*_0_ is given by Equation 4. The minority carrier diffusion length *L*_*P*_ in the four differently doped n-type silicon wafers was determined by surface photovoltage (SPV) measurements (see Supplementary Fig. S1 online). The extracted values are summarized in Table 1. *J*_0_ is calculated from Equation 4, using commonly accepted hole mobilities μ_*p*_^43^. *J*_0_ shows a clear decrease with increasing *N*_*D*_ from 8 to 0.04 pA/cm^2^ (cf. Table 2 and blue dots Fig. 7(b)). Following Equation 2, *V*_*oc*_ is calculated from *J*_0_. The blue dots in Fig. 7(a) show that the *V*_*oc*_ steadily increases from 563 mV to 696 mV with increasing doping concentration *N*_*D*_. The calculated *V*_*oc*_ shows the same dependence on *N*_*D*_ as the measured *V*_*oc*_ of the solar cells, indicating that the diffusion of minority carriers in an abrupt p^+^n-junction describes the transport properties clearly better than the thermionic emission of majority carriers over a Schottky junction. Even though the doping dependence of *V*_*oc*_ is well replicated, the very simple assumption of an ideally abrupt p^+^n-junction for the hybrid n-Si/PEDOT:PSS solar cell overestimates *V*_*oc*_ compared to the measured values.

**Figure 8:**
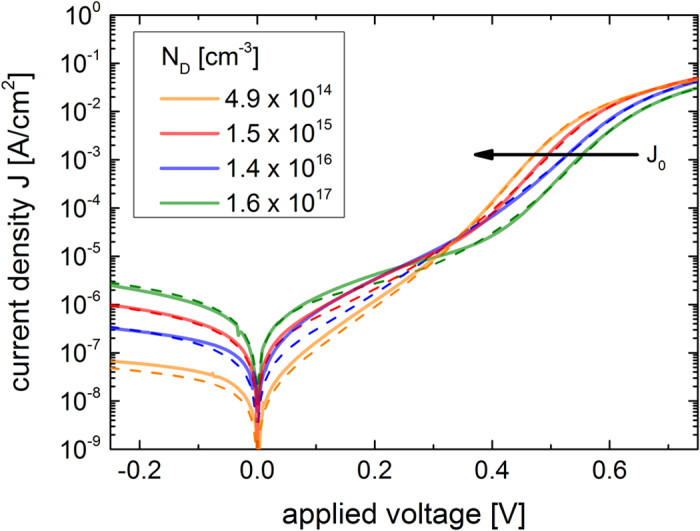
J-V characteristic of the n-Si/PEDOT:PSS interfaces. The dark J-V plots of n-Si/PEDOT:PSS solar cells are fitted by the two-diode model following Equation 6 (dashed lines). The arrow illustrates the increase of the dark saturation current density *J*_0_ with decrasing doping concentration *N*_*D*_.

The magnitude of calculated *J*_0_ for both junction models differ at least by a factor 100. To determine the dark saturation current density *J*_0_ of the prepared hybrid n-Si/PEDOT:PSS solar cells the dark current density-voltage (J-V) characteristics were measured, shown in Fig. 8. Deviating from the ideal diode law (Equation 1), the current density for high and very low forward bias is governed by the serial and parallel resistance of the device. In the mid forward bias range one typically determines *J*_0_ by linear extrapolation of the J-V curve to V = 0, as it is frequently done for polymer/Si devices^7^,^11^,^20^,^30^. The n-Si/PEDOT:PSS junction clearly shows a dependence of *J*_0_ on *N*_*D*_, indicated by the arrow in Fig. 8, as expected from Equation 4 for an abrupt p^+^n-junction. As mentioned before the extrapolation procedure assuming the simple Shockley equation overestimates the value of *J*_0_. Instead this non-ideal abrupt p^+^n-junction with silicon is better described by the two–diode model (Equation 6). This model includes area specific serial and parallel resistances as well as current contributions from diffusion and recombination in the bulk, and generation and recombination at defects in the space charge region. A least square routine was used to obtain the best fit of Equation 6 to the dark J-V curves (see Supplementary Fig. S2 online), shown as dashed lines in Fig. 8. The fits describe the trend of the dark J-V-curves of the n-Si/PEDOT:PSS solar cells quite well. The fit parameter *J*_01_, describing the dark saturation current density from the diffusion of minority carriers in the bulk, is extracted in Table 2 and presented in Fig. 7(b) (black dots). *J*_01_ decreases with increasing *N*_*D*_ from 13 to 0.3 pA/cm^2^. The fit parameters reproduce well the calculated *J*_0_ values if considering an ideal abrupt p^+^n-junction (blue dots), both in magnitude and *N*_*D*_ dependence. Compared to the calculated values assuming a Schottky junction (green dots), the extracted values for *J*_0_ are orders of magnitude smaller, leading to a clearly larger *V*_*oc*_. This difference in magnitude for *J*_0_ assuming a Schottky or pn-junction for Si/polymer solar cells has been pointed out by other groups for dark J-V measurements on one single solar cell^11^ or by estimations of the *V*_*oc*_ calculating the *J*_0_ with measured carrier lifetime^7^,^22^. The *N*_*D*_-dependence and magnitude of *J*_0_ and subsequently of *V*_*oc*_, shown in this work, confirm that the ruling transport mechanism at n-Si/PEDOT:PSS interface is not thermionic emission of majority carriers but diffusion of minorities in the silicon bulk. Hence, a n-Si/PEDOT:PSS solar cell should be described as a pn-heterojunction.

## Conclusion and Outlook

In this work we have combined capacitance-voltage measurements on hybrid n-Si/PEDOT:PSS junctions with photoelectron spectroscopy of PEDOT:PSS to develop a complete band diagram showing that silicon is strongly inverted at the interface to the polymer. This was verified for hybrid solar cells based on differently doped silicon wafers. By measuring and modeling the dark and illuminated current-voltage characteristics and comparing the extracted open-circuit voltage and dark saturation current density with values calculated from transport equations based on different junction models, we could show that only considering the strong inversion is an insufficient explanation for the high open-circuit voltages and promising efficiencies that are also observed in this work for n-Si/PEDOT:PSS solar cells. In fact we have demonstrated that the transport mechanism dominating the hybrid inorganic/organic n-Si/PEDOT:PSS junction is the diffusion of minority carriers in the silicon bulk and not the thermionic emission of majority carriers at the interface. With this we give a comprehensive explanation of the measured high open-circuit voltages and promising efficiencies for n-Si/PEDOT:PSS solar cells. This shows that hybrid n-Si/PEDOT:PSS solar cells should be described as abrupt p^+^n-heterojunctions and not as commonly done by Schottky junctions. In general, the results corroborate that hybrid inorganic/organic heterointerfaces can be great charge carrier selective contacts for photovoltaic and other opto-electronic devices.

## Methods

All devices were fabricated on planar n-type silicon <100> substrates, based on four wafers covering a doping concentration, *N*_*D*_, between 10^14^ cm^−3^ and 10^17^ cm^−3^. The minority carrier diffusion length, *L*_*P*_ (holes for n-Si), in the differently doped silicon was extracted by surface photovoltage (SPV) measurements on complete 4″ to 6″ wafers (see Supplementary online). Smaller samples (1.5 × 1.5 cm^2^) were cleaned by ultrasonification in acetone and isopropanol. To define and isolate the active area (1.17 cm^2^) the photoresist (nLof, Microchemicals) was spin-coated onto the samples and developed by UV lithography. The native oxide on the silicon surface was removed by hydrofluoric acid (5% HF for 30 s). PEDOT:PSS (PH1000, Heraeus Clevios) was filtered with a polyvinylidene fluoride membrane (0.45 μm porosity) to remove agglomerations. To increase the conductivity of the final film, 5 vol% DMSO were added to the PEDOT:PSS solution. Since PEDOT:PSS is a water based solution it was necessary to add a wetting agent (0.1 vol% FS31, Capstone) to ensure a proper interface formation on hydrophobic H-passivated silicon. PEDOT:PSS was spin coated at 2000 rpm for 10 s and subsequently annealed at 130 °C for 15 min under standard atmospheric conditions. The thickness of the polymer layer was approximately 115 nm as determined by ellipsometry.

The density of valence band states (DOVS) of PEDOT:PSS was probed by UPS. Therefore, polymer layers on silicon were transferred into the ultrahigh vacuum system (base pressure <5 × 10^−10^ mbar) immediately after complete fabrication. The UPS measurements were conducted using an excitation energy of 6.5 eV, provided by a high-pressure Xenon lamp and a double grating monochromator. A spot of approx. 10 mm^2^ was illuminated with an integration time of 40 s. Photoemission spectra were collected by an energy analyzer with a resolution of 125 meV. The kinetic energy of the detected photoelectrons was converted to binding energy (*E*_*bind*_) by calibrating the position of the Fermi level *E*_*F*_ (*E*_*bind*_ = 0) with a gold standard.

For complete photovoltaic devices an In/Ga eutectic was scratched into the silicon as a back contact and a gold grid (finger width 80 μm) was evaporated by an electron beam through a shadow mask on the polymer as a front contact. Fig. 1 shows a schematic of the n-Si/PEDOT:PSS device.

Electrical device characterization was carried out with a Keithley SCS 4200 semiconductor characterization system equipped with preamplifiers and a capacitance-voltage unit using a four-terminal configuration for contacting. capacitance-voltage (C-V) measurements were performed at 10 kHz with an ac amplitude of 10 mV and a voltage sweep between −2 V and 2 V. The built-in voltage of the hybrid n-Si/PEDOT:PSS junction as well as the doping concentration of the silicon wafer were obtained from the V-axis intercept and the slope of the linearly fitted data, respectively. Current density-voltage (J-V) characteristics were measured in the dark and under illumination. The dark J-V plots were analyzed by a numerical algorithm based on the two-diode model including area specific parallel and serial resistance (see Supplementary online). The saturation current density is extracted as a model parameter. To characterize the photovoltaic response of the devices, samples were irradiated through the transparent PEDOT:PSS layer by an AM1.5 reference spectrum (Oriel Sol3A Class AAA Solar Simulators, Newport). All standard solar cell parameters where derived from these illuminated J-V measurements.

## Additional Information

**How to cite this article**: Jäckle, S. *et al.* Junction formation and current transport mechanisms in hybrid n-Si/PEDOT:PSS solar cells. *Sci. Rep.* **5**, 13008; doi: 10.1038/srep13008 (2015).

## Supplementary Material

Supplementary Information

## Figures and Tables

**Table 1:** Summary of n-Si/PEDOT:PSS interface, solar cell and silicon substrate parameters for all differently doped n-type silicon wafers (all abbreviations are defined in the text).

Capacitance–Measurements			Solar cell parameter				SPV
N_D_ [cm^−3^]	ψ_bi_ [V]	*ϕ*_Bn_ [V]	V_oc_ [V]	J_sc_ [mA/cm^2^]	FF	PCE [%]	L_p_ [μm]
4.9 × 10^14^	0.672	0.96	0.542	31.6	0.64	11.0	332
1.5 × 10^15^	0.712	0.97	0.564	31.1	0.66	11.5	257
1.4 × 10^16^	0.803	0.95	0.608	31.3	0.73	13.8	187
1.6 × 10^17^	0.878	0.94	0.634	29.1	0.75	13.9	120

**Table 2:** Summary of open circuit voltage *V*_*oc*_ and saturation current density *J*_0_ extracted from the illuminated and dark J-V-curves (Figs 2 and 8) as well as calculated assuming a Schottky junction (Eq. 3) and an abrupt p^+^n-junction (Eq. 4) for different silicon substrate doping (*N*_*D*_).

N_D_ [cm^−3^]	J-V-curves		Schottky junction		p^+^n-junction	
	J_01_ [A/cm^2^]	*V*_*oc*_ [V]	J_0_ [A/cm^2^]	*V*_*oc*_ [V]	J_0_ [A/cm^2^]	*V*_*oc*_ [V]
4.9 × 10^14^	1.3 **×** 10^−11^	0.542	9.7 **×** 10^−10^	0.441	8.0 **×** 10^−12^	0.563
1.5 × 10^15^	3.1 **×** 10^−12^	0.564	6.8 **×** 10^−10^	0.450	3.4 **×** 10^−12^	0.585
1.4 × 10^16^	4.5 **×** 10^−13^	0.608	3.1 **×** 10^−10^	0.470	4.4 **×** 10^−13^	0.637
1.6 × 10^17^	3.2 **×** 10^−13^	0.634	4.6 **×** 10^−10^	0.458	4.1 **×** 10^−14^	0.696
