# Supplementary material for: Junction formation and current transport mechanisms in hybrid n-Si/PEDOT:PSS solar cells
Source: Sci Rep. 2015 Aug 17;5:13008. doi: 10.1038/srep13008 (PMC4538380; doi:10.1038/srep13008)
Supplement: Supplementary Information [file srep13008-s1.pdf]

Supplementary Information

Junction formation and current transport mechanisms in hybrid n-Si/PEDOT:PSS solar cells

Sara Jäckle*, Matthias Mattiza, Martin Liebhaber, Gerald Brönstrup, Mathias Rommel, Klaus Lips, Silke Christiansen

Surface Photovoltage (SPV)

The minority carrier diffusion length L_p (for holes) of the differently n-doped silicon wafers was determined by surface photovoltage measurements on full wafers (diameter 4″ - 6″, thickness 525 - 615 µm). SPV was chosen because for this method the measurement is not influenced by surface recombination at the wafer frontside (illuminated side). All measurements were performed using a WT-2500 Semilab SPV system using eight excitation wavelengths with an 8 mm Kelvin probe head. Thus, points close to the wafer edge cannot be correctly measured.

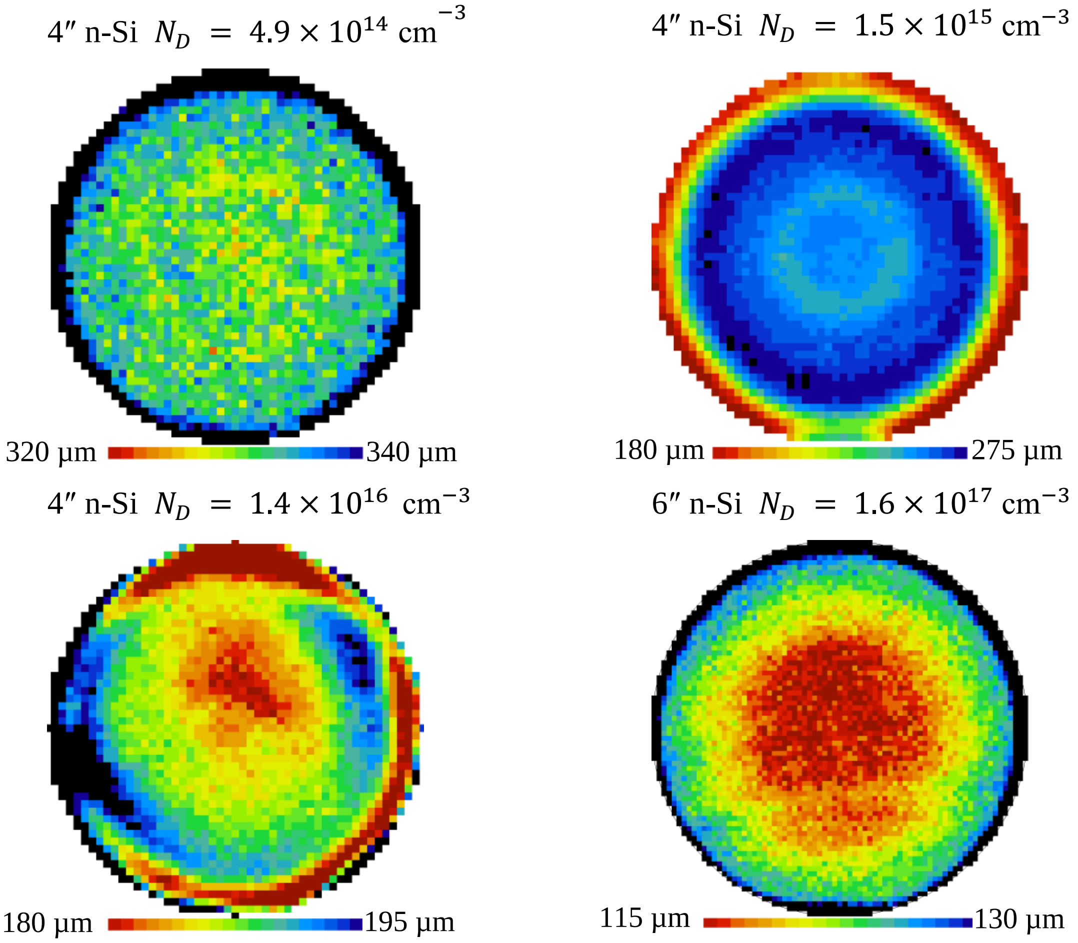

Figure S1. Diffusion length maps of differently doped whole silicon wafers. Minority carrier diffusion length L_P depicted in a 2 mm raster by a colour code.

The diffusion length collected in **Table 1** of the paper was obtained by averaging over the 2 mm raster with an edge exclusion of 5 mm. Very high regression coefficients were obtained for the SPV results (minimum value of 0.995 for the sample with the smallest doping concentration) which indicates that the obtained diffusion length values are reliable.

Fitting algorithm based on the two-diode model

The two-diode model is commonly used to describe the current density-voltage (J-V) characteristic of solar cells based on an indirect semiconductor. It includes the diffusion current in the bulk and the recombination current in the space charge region and is described by **Equation 6** in the paper.[1] For a diode measured in the dark it reduces to:

$$J = J_{01}\left(exp\left(\frac{q(\mathrm{V} - R_s J)}{kT}\right) - 1\right) + J_{02}\left(\exp\left(\frac{q(\mathrm{V} - R_s J)}{2kT}\right) - 1\right) + \frac{\mathrm{V} - R_s J}{R_p}$$

This implicit equation is analytically not solvable. Therefore, iterative methods are used to extract the four model parameters: saturation current density for diffusion J_{01} and recombination J_{02}, area specific shunt resistance R_p and series resistance R_s. The dark J-V-curves of the hybrid n-Si/PEDOT:PSS solar cells are numerically fitted with the Levenberg-Marquardt algorithm for the equations based on Hovinen.[2] The relative weighted deviation $\frac{|J_{\mathrm{exp}} - J_{\mathrm{calc}}|}{|J_{\mathrm{exp}}|}$ of the experimental current densities J_{exp} and the calculated J_{calc} was minimized. A large range of starting parameters was used and the best fits were chosen. Here, the best fits are those with the smallest sum of the squared deviations scaled with the squared value of the experimental values and are collected in Table S1. In Figure S2 the measured (blue) and fitted (red) curves are shown for the solar cells based on the differently doped silicon substrates. Table S1 also summarizes the extracted model parameters.

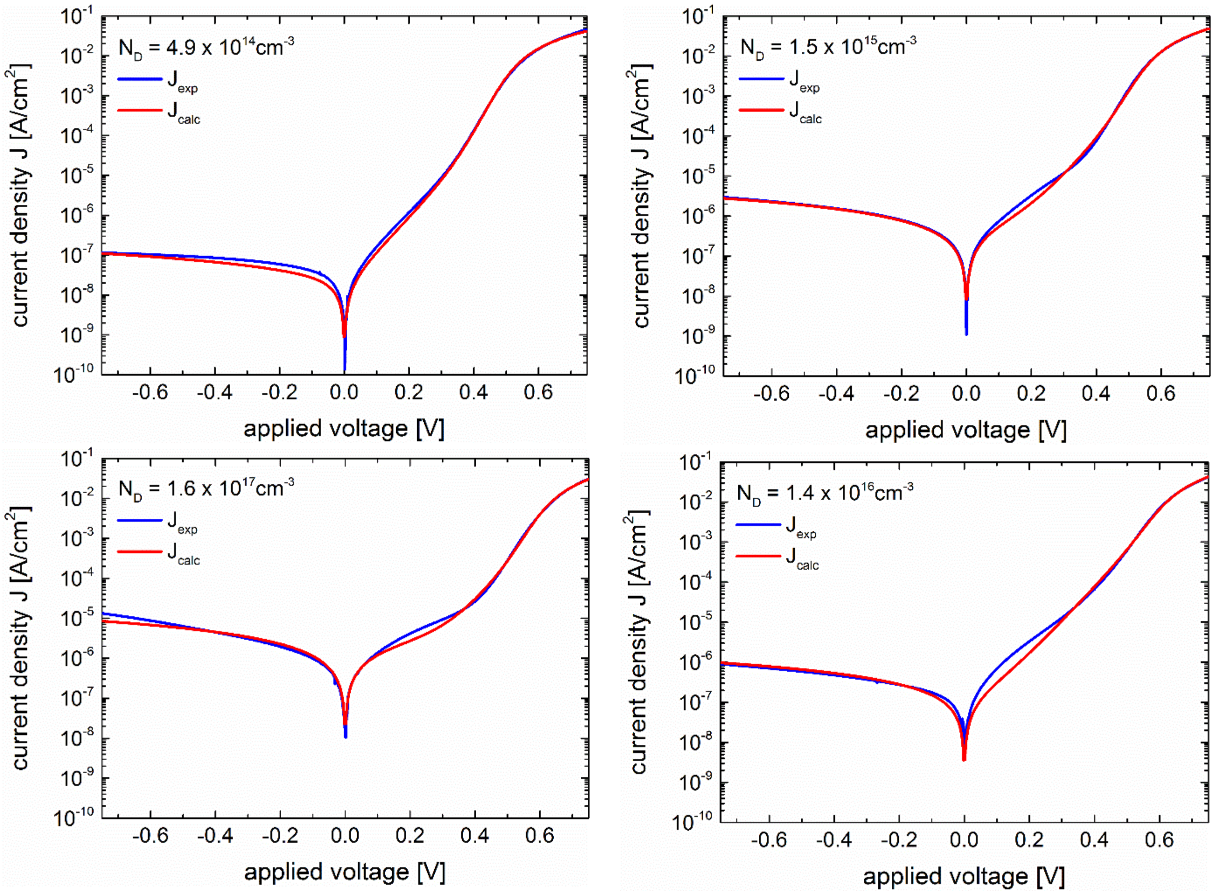

Figure S2. Dark current density - voltage characteristics of n-Si/PEDOT:PSS solar cells. Experiementaly determined (J_{exp}) and calculated (J_{calc}) current densities of devices based on differently doped (N_D) silicon substrates.

N_D [cm^{-3}]	J_{01} [A/cm^2]	J_{02} [A/cm^2]	R_p [Ωcm^2]	R_s [Ωcm^2]	$\frac{1}{N}\sum\frac{\lvert J_{exp}-J_{calc}\rvert^2}{\lvert J_{exp}\rvert^2}$
**4.9 x 10^{14}**	1.3 x 10^{-11}	1.7 x 10^{-8}	8.0 x 10^{6}	4.6	8.73 x 10^{-2}
**1.5 x 10^{15}**	3.1 x 10^{-12}	2.8 x 10^{-8}	2.7 x 10^{5}	3.1	2.09 x 10^{-2}
**1.4 x 10^{16}**	4.5 x 10^{-13}	2.9 x 10^{-8}	7.9 x 10^{5}	2.6	5.85 x 10^{-2}
**1.6 x 10^{17}**	3.2 x 10^{-13}	9.4 x 10^{-9}	8.8 x 10^{4}	3.6	3.64 x 10^{-2}

Table S1. Summary of the model parameters extracted from the fitted curves in Figure S2. All abriviations are defined in the text.

References

1. Sze, S. M. & Ng, K. K. *Physics of Semiconductor Devices*. (Wiley, 2006).
2. Hovinen, A. Fitting of the solar cell IV-curve to the two diode model. *Physica Scripta* **T54,** 175–176 (1994).
